# TACE-HAIC plus TKIs with/without PD-1 inhibitors for unresectable hepatocellular carcinoma: a propensity score matching study

**DOI:** 10.3389/fonc.2026.1874177

**Published:** 2026-08-14

**Authors:** Xiadi Weng, Yang Zhou, Guoxu Fang, Yongyi Zeng

**Affiliations:** 1 Department of Hepatobiliary Surgery, First Affiliated Hospital of Fujian Medical University, Fuzhou, China; 2 Department of Interventional Radiology, Mengchao Hepatobiliary Hospital of Fujian Medical University, Fuzhou, China; 3 Department of Hepatopancreatobiliary Surgery, Mengchao Hepatobiliary Hospital of Fujian Medical University, Fuzhou, China

**Keywords:** hepatic arterial infusion chemotherapy, hepatocellular carcinoma, lenvatinib, PD-1 inhibitors, transarterial chemoembolization

## Abstract

**Background and aims:**

Patients with unresectable hepatocellular carcinoma (uHCC) have a poor prognosis, and effective systemic treatment options remain limited. We aimed to evaluate the safety and efficacy of adding programmed death-1 (PD-1) inhibitors to transarterial chemoembolization combined with hepatic arterial infusion chemotherapy (TACE-HAIC)+lenvatinib.

**Methods:**

We retrospectively analyzed data from patients with uHCC who received TACE-HAIC+lenvatinib, without or with PD-1 inhibitors (n = 124). Patients were stratified into a triple-therapy group (TACE-HAIC+lenvatinib; n = 71) and a quadruple-therapy group (TACE-HAIC+lenvatinib+PD-1 inhibitors; n = 53), based on whether PD-1 inhibitors were included in the first-line regimen. Overall survival (OS), progression-free survival (PFS), and adverse event rate were compared between the groups. Propensity score matching was performed to reduce confounding bias.

**Results:**

After propensity score matching (PSM), 43 matched pairs were identified. The median OS was 11.0 months [95% confidence interval (CI), 10.0–16.0] in the triple-therapy group and 25.0 months (95% CI: 12.0–not reached) in the quadruple-therapy group. The median PFS was 8.6 months (95% CI: 6.37–11.77) and 12.1 months (95% CI: 8.7–not reached), respectively. Both OS and PFS were significantly improved in the quadruple-therapy group (P < 0.05).

**Conclusions:**

The addition of PD-1 inhibitors to TACE-HAIC plus lenvatinib significantly improved both OS and PFS without a statistically significant increase in adverse events. However, careful monitoring and prompt management of immune-related adverse events remain warranted during treatment.

## Introduction

Hepatocellular carcinoma (HCC) is a major global health burden and the third leading cause of cancer-related mortality worldwide (1). Radical surgical resection remains the treatment of choice for early-stage disease (2). However, due to the insidious onset of HCC, most patients are diagnosed at an advanced stage, rendering them ineligible for curative surgery (3, 4).

Transarterial chemoembolization (TACE) and hepatic arterial infusion chemotherapy (HAIC) play established roles in the management of advanced HCC (5). In patients with unresectable HCC (uHCC), accumulating evidence indicates that the combination of HAIC and TACE provides superior therapeutic outcomes compared with TACE alone (6, 7). Nevertheless, arterial embolization can aggravate tumor ischemia and hypoxia, thereby creating a microenvironment that may paradoxically promote tumor progression and facilitate recurrence and metastasis (8, 9). Small-molecule antiangiogenic agents, particularly tyrosine kinase inhibitors (TKIs), exert antitumor effects by inhibiting vascular endothelial growth factor receptor signaling. Consequently, TACE and HAIC are frequently combined with TKIs to suppress tumor angiogenesis and improve survival outcomes (10).

Despite advances in multidisciplinary team (MDT) management and precision medicine, the aforementioned triple-therapy strategy (TACE-HAIC+TKIs) remains insufficient to achieve optimal long-term survival in patients with uHCC (11, 12). Therefore, more effective and safe combination regimens are urgently needed. In recent years, immune checkpoint inhibitors have emerged as a promising therapeutic option for uHCC (13). These agents can reverse tumor-induced immune suppression and may convert the local tumor antigen release induced by locoregional therapies into a systemic and durable antitumor immune response, thereby enhancing survival (14). However, the safety and efficacy of quadruple therapy combining TACE-HAIC, TKIs, and programmed death-1 (PD-1) inhibitors have not been fully elucidated.

In this retrospective study, we evaluated the efficacy and safety of TACE-HAIC combined with lenvatinib, with or without PD-1 inhibitors, in patients with uHCC. We focused on clinically meaningful real-world outcomes, including tumor response, PFS, and OS, to better characterize the potential benefits of incorporating PD-1 inhibitors into this treatment strategy.

## Methods

### Patients

This retrospective study included 124 consecutive patients with initially uHCC who received TACE-HAIC+lenvatinib, without or with PD-1 inhibitors, between January 2020 and December 2023 from Mengchao Hepatobiliary Hospital of Fujian Medical University. The study was conducted in accordance with the Declaration of Helsinki and was approved by the institutional review board (No. 2023-095-01). Written informed consent was obtained from all patients at the time of the final follow-up.

HCC was diagnosed based on histological, cytological, or clinical criteria in accordance with the European Association for the Study of the Liver and the American Association for the Study of Liver Diseases guidelines (15, 16). Tumor unresectability was determined through MDT evaluation and classified as intermediate- or advanced-stage HCC. The inclusion criteria were as follows: (a) age ≥18 years; (b) receipt of TACE-HAIC+lenvatinib, without or with PD-1 inhibitors, as first-line treatment; (c) Child–Pugh class A or B liver function; (d) Eastern Cooperative Oncology Group performance status of 0–1; (e) adequate hematologic function; and (f) at least one measurable lesion suitable for tumor burden assessment according to the modified Response Evaluation Criteria in Solid Tumors (mRECIST) and Response Evaluation Criteria in Solid Tumors version 1.1 (RECIST v1.1) (17).

The exclusion criteria were as follows: (a) severe cardiac, pulmonary, or renal comorbidities; (b) history of another primary malignancy; (c) prior locoregional or systemic anticancer therapy; (d) concurrent malignancies; (e) autoimmune disease requiring active treatment or known immunodeficiency; and (f) incomplete essential clinical data.

### TACE-HAIC procedure

All patients underwent TACE-HAIC. Under local anesthesia, vascular access was obtained via the right femoral artery using the Seldinger technique. Angiography of the common hepatic artery or celiac trunk was performed to identify tumor burden, including lesion number, size, location, and feeding arteries. A microcatheter was then superselectively advanced into the tumor-feeding arteries, through which an emulsion of iodized oil (5–20 mL) and epirubicin (30 mg/m^2^) was administered. Subsequently, the catheter was positioned in the tumor-feeding artery for FOLFOX-based chemotherapy, consisting of oxaliplatin (85 mg/m^2^ over 2 h), calcium levofolinate (400 mg/m^2^ over 2 h), a bolus of 5-fluorouracil (5-FU; 400 mg/m^2^), followed by continuous infusion of 5-FU (2400 mg/m^2^ over 46 h). TACE-HAIC was repeated every 4–6 weeks based on tumor response (particularly residual arterial blood supply) and hepatic functional reserve.

### TKIs and PD-1 inhibitors

The TKI used in this study was lenvatinib, administered orally once daily at a dose of 12 mg for patients with a body weight of ≥60 kg or 8 mg for those with a body weight of <60 kg. Patients in the triple-therapy group received TACE-HAIC plus lenvatinib without PD-1 inhibitors. In the quadruple-therapy group, PD-1 inhibitors included camrelizumab and tislelizumab. The selection of PD-1 inhibitors was based on drug cost, availability through charitable access programs, local health insurance reimbursement policies, and patient preference (**Supplementary Table 1**). PD-1 inhibitors were used 200 mg of camrelizumab, or 200 mg of tislelizumab, every three weeks (**Supplementary Table 1**). Treatment with lenvatinib and PD-1 inhibitors was initiated within 3–14 days after TACE-HAIC, depending on the patient’s general condition and recovery of liver function, and it was continued until disease progression or the occurrence of unacceptable treatment-related toxicity.

### Antiviral therapy was administered to all patients with hepatitis B virus infection.

#### Efficacy and safety assessment

Tumor response was evaluated 4–6 weeks after initiation of treatment using contrast-enhanced abdominal computed tomography (CT) or magnetic resonance imaging, along with chest CT. Radiological assessments were independently performed by two experienced radiologists in accordance with RECIST v1.1 and mRECIST. Responses were categorized as complete response (CR), partial response (PR), stable disease (SD), or progressive disease (PD). Time to response was defined as the interval from initiation of triple or quadruple therapy to the first documented CR or PR. The best overall response during treatment was recorded. The primary endpoints were OS and PFS. OS was defined as the time from initiation of transarterial therapy to death from any cause. PFS was defined as the time from treatment initiation to disease progression or death, whichever occurred first. Disease progression was determined based on contrast-enhanced CT or magnetic resonance imaging according to mRECIST criteria. Secondary endpoints included objective response rate (ORR) and disease control rate (DCR). ORR was defined as the proportion of patients achieving CR or PR, sustained for at least 4 weeks after initial radiological confirmation. DCR was defined as the proportion of patients achieving CR, PR, or SD.

Safety was assessed based on physical examinations, vital signs, hematologic and biochemical laboratory parameters, and the incidence of adverse events (AEs), recorded from treatment initiation through 30 days after the last dose or study completion. Treatment-related AEs were graded according to the National Cancer Institute Common Terminology Criteria for Adverse Events, version 5.0 (18).

### Data collection and follow-up

Baseline clinical data were collected from the electronic medical records, including age, sex, Eastern Cooperative Oncology Group (ECOG) performance status, HBV infection status, Barcelona Clinic Liver Cancer (BCLC) stage, Child–Pugh class, tumor size and number, vascular invasion, extrahepatic metastasis, and laboratory parameters. Laboratory assessments, including complete blood count, serum biochemistry, and HCC-related tumor markers [alpha-fetoprotein (AFP) and protein induced by vitamin K absence or antagonist-II (PIVKA-II)], were performed before and after each treatment cycle. Radiological examinations were conducted regularly to evaluate treatment response and disease progression. Patients were followed until death or the study cutoff date (May 1, 2025), whichever occurred first.

Patients who were not eligible for surgical resection continued combination therapy until disease progression or intolerable toxicity.

For patients undergoing surgical resection, lenvatinib was withheld for 1 week and PD-1 inhibitors for 4 weeks before and after surgery. Postoperative systemic therapy with lenvatinib and PD-1 inhibitors was continued for 3–12 months, based on pathological findings, radiologic assessment, and patient tolerance. After resection, the patients were followed at 1-month postoperatively, every 3–4 months during the first 2 years, and every 4–6 months thereafter.

If disease progression occurred during follow-up, subsequent treatment strategies were determined through MDT discussion with informed patient consent. Available options included iodine-125 seed implantation, external beam radiotherapy, tumor ablation, additional immunotherapy, or alternative targeted therapies (**Supplementary Table 2**).

### PSM

PSM was performed to reduce selection bias and balance baseline characteristics between the triple-therapy and quadruple-therapy groups. Propensity scores were estimated using a multivariable logistic regression model incorporating age, sex, ECOG performance status, Child–Pugh class, AFP level, tumor size, tumor number, macrovascular invasion, extrahepatic metastasis, BCLC stage, and other baseline variables shown in **Table 1**. Patients were matched in a 1:1 ratio using nearest-neighbor matching without replacement with a caliper width of 0.15.

**Table 1:** Baseline characteristics of the study cohort before and after PSM.

Variable	Before PSM					After PSM					
	Total (n = 124)	Triple Therapy (n = 71)	Quadruple therapy (n = 53)	Statistic	P	Total (n = 86)		Triple therapy (n = 43)	Quadruple therapy (n = 43)	Statistic	P
Gender, n (%)				χ^2^=0.209	0.648					χ^2^=0.179	0.672
Female	9 (7.26)	4 (5.63)	5 (9.43)			6 (6.98)		2 (4.65)	4 (9.30)		
Male	115 (92.74)	67 (94.37)	48 (90.57)			80 (93.02)		41 (95.35)	39 (90.70)		
Age, n (%)				χ^2^=0.001	0.981					χ^2^=0.047	0.829
≥55 years	68 (54.84)	39 (54.93)	29 (54.72)			47 (54.65)		24 (55.81)	23 (53.49)		
<55 years	56 (45.16)	32 (45.07)	24 (45.28)			39 (45.35)		19 (44.19)	20 (46.51)		
ECOGs, n (%)				χ^2^=2.059	0.151					χ^2^=0.187	0.665
0	54 (43.55)	27 (38.03)	27 (50.94)			40 (46.51)		19 (44.19)	21 (48.84)		
1	70 (56.45)	44 (61.97)	26 (49.06)			46 (53.49)		24 (55.81)	22 (51.16)		
Child–Pugh, n (%)				χ^2^=1.748	0.186					χ^2^=0.091	0.763
A	101 (81.45)	55 (77.46)	46 (86.79)			73 (84.88)		37 (86.05)	36 (83.72)		
B	23 (18.55)	16 (22.54)	7 (13.21)			13 (15.12)		6 (13.95)	7 (16.28)		
HBV, n (%)				χ^2^=0.260	0.610					χ^2^=0.262	0.609
Absent	11 (8.87)	5 (7.04)	6 (11.32)			4 (4.65)		1 (2.33)	3 (6.98)		
Present	113 (91.13)	66 (92.96)	47 (88.68)			82 (95.35)		42 (97.67)	40 (93.02)		
Imaging cirrhosis, n (%)				χ^2^=2.956	0.086					χ^2^=0.068	0.795
Absent	24 (19.35)	10 (14.08)	14 (26.42)			19 (22.09)		9 (20.93)	10 (23.26)		
Present	100 (80.65)	61 (85.92)	39 (73.58)			67 (77.91)		34 (79.07)	33 (76.74)		
Ascites, n (%)				χ^2^=1.042	0.307					χ^2^=0.047	0.828
Absent	72 (58.06)	44 (61.97)	28 (52.83)			49 (56.98)		25 (58.14)	24 (55.81)		
Present	52 (41.94)	27 (38.03)	25 (47.17)			37 (43.02)		18 (41.86)	19 (44.19)		
Main tumor size, n (%)				χ^2^=2.297	0.130					χ^2^=0.000	1.000
≥10 cm	42 (33.87)	28 (39.44)	14 (26.42)			24 (27.91)		12 (27.91)	12 (27.91)		
<10 cm	82 (66.13)	43 (60.56)	39 (73.58)			62 (72.09)		31 (72.09)	31 (72.09)		
Tumor number, n (%)				χ^2^=1.748	0.186					χ^2^=0.000	1.000
Single	23 (18.55)	16 (22.54)	7 (13.21)			14 (16.28)		7 (16.28)	7 (16.28)		
Multiple	101 (81.45)	55 (77.46)	46 (86.79)			72 (83.72)		36 (83.72)	36 (83.72)		
Tumor involvement, n (%)				χ^2^=3.625	0.057					χ^2^=0.000	1.000
Hemiliver	77 (62.10)	39 (54.93)	38 (71.70)			58 (67.44)		29 (67.44)	29 (67.44)		
Both hemilivers	47 (37.90)	32 (45.07)	15 (28.30)			28 (32.56)		14 (32.56)	14 (32.56)		
Macrovascular invasion, n (%)				χ^2^=0.000	0.989					χ^2^=0.281	0.596
Absent	28 (22.58)	16 (22.54)	12 (22.64)			18 (20.93)		8 (18.60)	10 (23.26)		
Present	96 (77.42)	55 (77.46)	41 (77.36)			68 (79.07)		35 (81.40)	33 (76.74)		
Extrahepatic metastasis, n (%)				χ^2^=1.900	0.168					χ^2^=0.847	0.357
Absent	92 (74.19)	56 (78.87)	36 (67.92)			58 (67.44)		31 (72.09)	27 (62.79)		
Present	32 (25.81)	15 (21.13)	17 (32.08)			28 (32.56)		12 (27.91)	16 (37.21)		
BCLC stage, n (%)				χ^2^=0.851	0.356					χ^2^=0.000	1.000
B	13 (10.48)	9 (12.68)	4 (7.55)			8 (9.30)		4 (9.30)	4 (9.30)		
C	111 (89.52)	62 (87.32)	49 (92.45)			78 (90.7)		39 (90.70)	39 (90.70)		
Hemoglobin, n (%)				χ^2^=1.142	0.285					χ^2^=0.091	0.763
<110 g/L	19 (15.32)	13 (18.31)	6 (11.32)			13 (15.12)		7 (16.28)	6 (13.95)		
≥110 g/L	105 (84.68)	58 (81.69)	47 (88.68)			73 (84.88)		36 (83.72)	37 (86.05)		
PLT, n (%)				χ^2^=0.000	1.000					χ^2^=1.613	0.204
≥100 × 10^9^/L	11 (8.87)	6 (8.45)	5 (9.43)			6 (6.98)		1 (2.33)	5 (11.63)		
<100 × 10^9^/L	113 (91.13)	65 (91.55)	48 (90.57)			80 (93.02)		42 (97.67)	38 (88.37)		
ALB, n (%)				χ^2^=0.166	0.684					χ^2^=0.819	0.365
≥35 g/L	47 (37.90)	28 (39.44)	19 (35.85)			30 (34.88)		13 (30.23)	17 (39.53)		
<35 g/L	77 (62.10)	43 (60.56)	34 (64.15)			56 (65.12)		30 (69.77)	26 (60.47)		
TBIL, n (%)				χ^2^=1.134	0.287					χ^2^=0.847	0.357
>17.1 umol/L	80 (64.52)	43 (60.56)	37 (69.81)			58 (67.44)		31 (72.09)	27 (62.79)		
≤17.1 umol/L	44 (35.48)	28 (39.44)	16 (30.19)			28 (32.56)		12 (27.91)	16 (37.21)		
ALT, n (%)				χ^2^=0.113	0.736					χ^2^=0.000	1.000
≥40 U/L	54 (43.55)	30 (42.25)	24 (45.28)			38 (44.19)		19 (44.19)	19 (44.19)		
<40 U/L	70 (56.45)	41 (57.75)	29 (54.72)			48 (55.81)		24 (55.81)	24 (55.81)		
AST, n (%)				χ^2^=0.096	0.756					χ^2^=0.608	0.436
≥40 U/L	25 (20.16)	15 (21.13)	10 (18.87)			19 (22.09)		11 (25.58)	8 (18.60)		
<40 U/L	99 (79.84)	56 (78.87)	43 (81.13)			67 (77.91)		32 (74.42)	35 (81.40)		
AFP, n (%)				χ^2^=0.257	0.612					χ^2^=0.049	0.825
≥400 ng/mL	50 (40.32)	30 (42.25)	20 (37.74)			33 (38.37)		16 (37.21)	17 (39.53)		
<400 ng/mL	74 (59.68)	41 (57.75)	33 (62.26)			53 (61.63)		27 (62.79)	26 (60.47)		

*P* values were calculated using a two-sided χ^2^ test; when the sample size was small, and the expected frequencies were <5, Fisher’s exact test was applied.Abbreviations: AFP, alpha-fetoprotein; ALB, albumin; ALT, alanine aminotransferase; AST, aspartate aminotransferase; BCLC, Barcelona Clinic for Liver Cancer; ECOG, Eastern Cooperative Oncology Group performance status; HBV, hepatitis B virus; PLT, platelet; TBIL, total bilirubin; uHCC, unresectable hepatocellular carcinoma.

### Statistical analysis

Continuous variables were compared using independent-samples or paired-samples t-tests. Categorical variables were compared using the χ^2^ test between the two groups; when expected cell counts were < 5, Fisher’s exact test was applied. OS and PFS were estimated using the Kaplan–Meier method and compared between groups using the log-rank test. All statistical tests were two-sided, and a P < 0.05 was considered to be statistically significant. All statistical analyses were performed using R (version 4.4.2, https://www.R-project.org/).

## Results

### Patient characteristics

**Figure 1:**
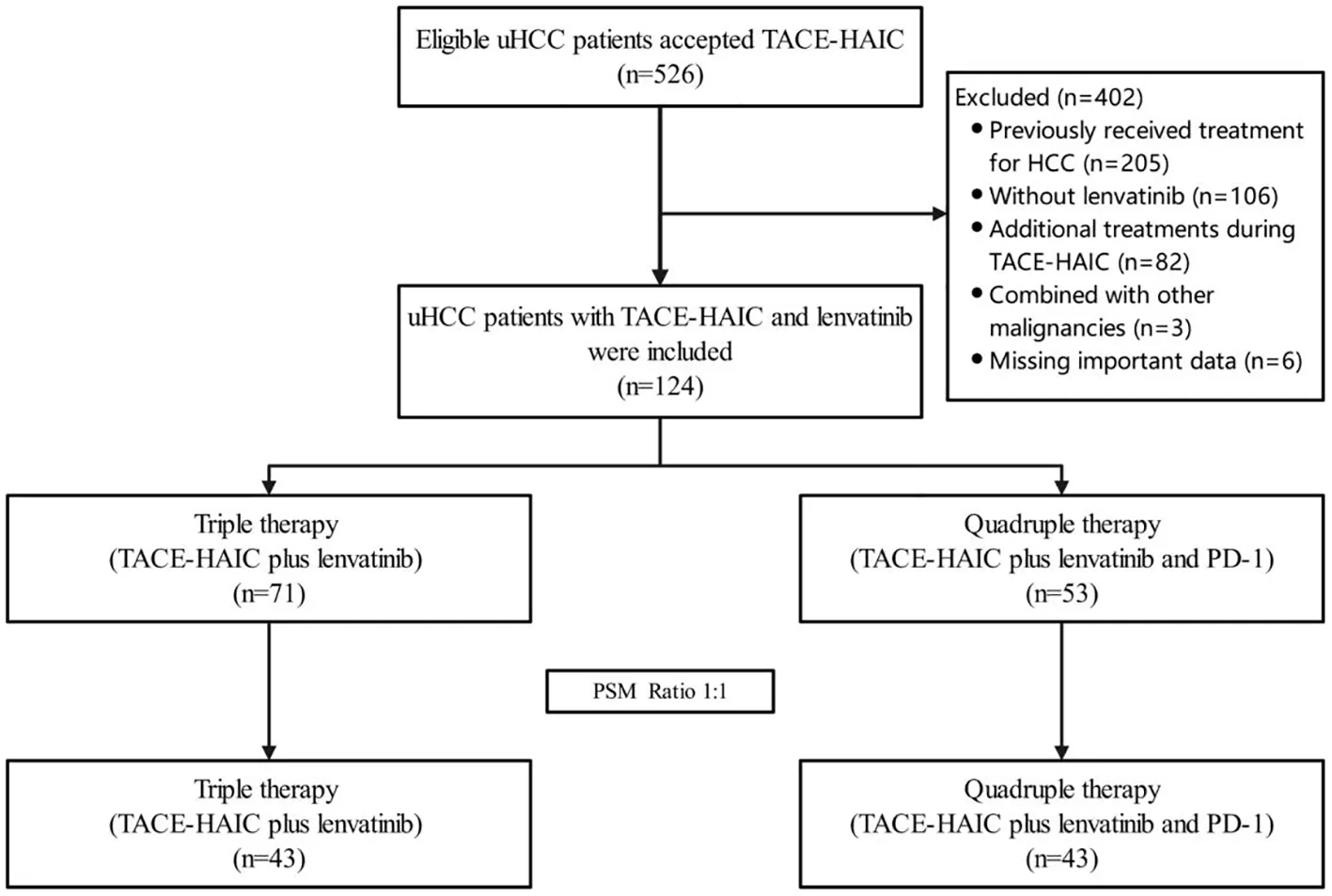
Flowchart depicting the study protocol. uHCC, unresectable hepatocellular carcinoma; TACE, transcatheter arterial chemoembolization; HAIC, hepatic artery infusion chemotherapy; PD-1, programmed death receptor-1.

The study flowchart is presented in **Figure 1**. A total of 526 patients with uHCC were screened for eligibility. After PSM, 86 patients were included in the matched cohort, comprising 43 patients in the triple-therapy group and 43 patients in the quadruple-therapy group. Baseline characteristics were well balanced between the two groups after matching, with no statistically significant differences observed (**Table 1**).

### Tumor responses

**Table 2** summarizes the best tumor responses before PSM. According to mRECIST, the ORR and DCR were 85.5% and 89.5%, respectively; according to RECIST v1.1, the corresponding rates were 40.3% and 89.5%.

**Table 2:** Treatment efficacy evaluated by RECIST v1.1 and mRECIST criteria before PSM.

	Total (n = 124)	Triple therapy (n = 71)	Quadruple therapy (n = 53)
RECIST v1.1 criteria, n (%)			
CR	1 (0.81)	0 (0.00)	1 (1.89)
PR	49 (39.52)	26 (36.62)	23 (43.40)
SD	61 (49.19)	37 (52.11)	24 (45.28)
PD	13 (10.48)	8 (11.27)	5 (9.43)
mRECIST criteria, n (%)			
CR	5 (4.03)	3 (4.23)	2 (3.77)
PR	101 (81.45)	56 (78.87)	45 (84.91)
SD	5 (4.03)	4 (5.63)	1 (1.89)
PD	13 (10.48)	8 (11.27)	5 (9.43)

CR, Complete response; mRECIST, Modified response evaluation criteria in solid tumors; PD, Progressive disease; PR, Partial response; RECIST, Response evaluation criteria in solid tumors; SD, Stable disease.

After PSM, the best tumor responses in the matched cohort are presented in **Table 3**. Based on mRECIST, the triple-therapy and quadruple-therapy groups showed comparable ORR (86.0% vs. 88.4%) and identical DCR (90.7% vs. 90.7%). Similarly, according to RECIST v1.1, there were no significant differences between the two groups in ORR (32.6% vs. 46.5%) or DCR (90.7% vs. 90.7%).

**Table 3:** Treatment efficacy evaluated by RECIST v1.1 and mRECIST criteria after PSM.

	Total (n = 86)	Triple therapy (n = 43)	Quadruple therapy (n = 43)
RECIST v1.1 criteria, n (%)			
CR	1 (1.16)	0 (0.00)	1 (2.33)
PR	33 (38.37)	14 (32.56)	19 (44.19)
SD	44 (51.16)	25 (58.14)	19 (44.19)
PD	8 (9.30)	4 (9.30)	4 (9.30)
mRECIST criteria, n (%)			
CR	2 (2.33)	1 (2.33)	1 (2.33)
PR	73 (84.88)	36 (83.72)	37 (86.05)
SD	3 (3.49)	2 (4.65)	1 (2.33)
PD	8 (9.30)	4 (9.30)	4 (9.30)

CR, Complete response; mRECIST, Modified response evaluation criteria in solid tumors; PD, Progressive disease; PR, Partial response; RECIST, Response evaluation criteria in solid tumors; SD, Stable disease.

### Survival outcomes

**Figure 2:**
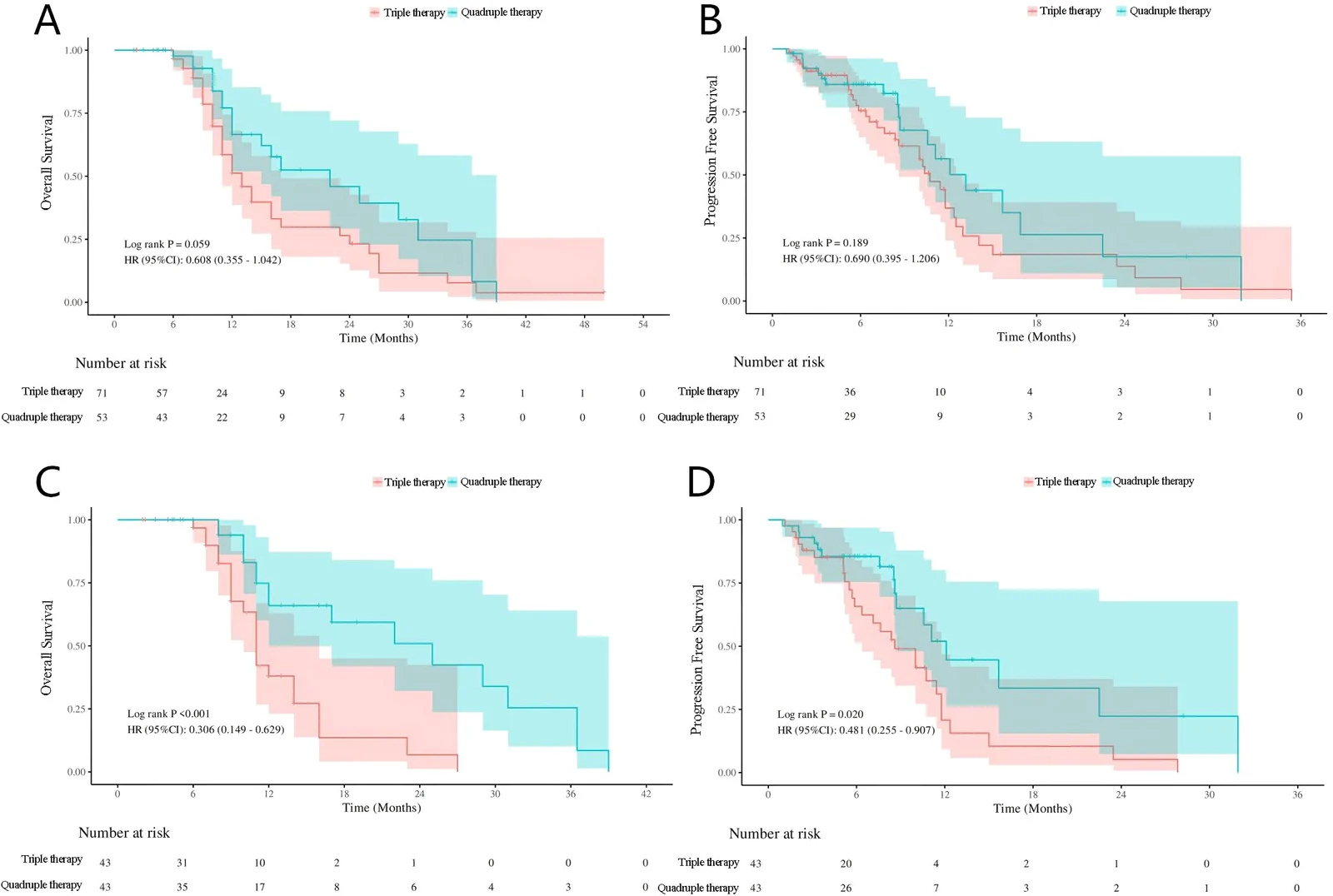
Kaplan–Meier survival curves comparing OS and PFS. **(A)** OS and **(B)** PFS of the patients who underwent the quadruple therapy versus triple therapy before PSM. **(C)** OS and **(D)** PFS of patients receiving quadruple therapy versus triple therapy after PSM. OS, Overall survival; PFS, Progression-free survival; PSM, Propensity score matching.

By the end of the follow-up period, 66 of 124 (53.2%) patients had died. Before PSM, the median follow-up duration was 21.6 months. In the unmatched cohort, the median OS was 22.0 months in the quadruple-therapy group and 13.0 months in the triple-therapy group, with a marginally significant difference between groups [hazard ratio (HR): 0.608; 95% confidence interval (CI): 0.355–1.042; P = 0.059] (**Figure 2A**). The median PFS was 13.17 months in the quadruple-therapy group and 10.73 months in the triple-therapy group, with no statistically significant difference (HR: 0.690; 95% CI: 0.395–1.206; P = 0.189) (**Figure 2B**). After PSM, the median OS was significantly longer in the quadruple-therapy group than in the triple-therapy group (25.0 vs. 11.0 months; HR: 0.306; 95% CI: 0.149–0.629; P < 0.001) (**Figure 2C**). Similarly, the quadruple-therapy group demonstrated a significant improvement in PFS (12.1 vs. 8.6 months; HR: 0.481; 95% CI: 0.255–0.907; P = 0.020) (**Figure 2D**).

### Subgroup analyses

Subgroup analyses revealed no significant intergroup survival differences, with no obvious population subgroups that derived extra benefits from quadruple therapy (**Supplementary Figure 1**).

### Treatment safety

Overall, 108 of 124 (87.1%) patients experienced treatment-emergent adverse events (TEAEs) following combination therapy. No significant differences were detected in TEAEs between the triple-therapy and quadruple-therapy groups (84.5% vs. 90.6%, P = 0.319). The most common TEAEs included abnormal liver function (50.7% vs. 50.9%), fever (46.5% vs. 52.8%), abdominal pain (42.3% vs. 45.3%), and anorexia (32.4% vs. 30.2%), with no significant between-group differences (all P > 0.05). Finally, 35 (28.2%) of 124 patients experienced grades 3–4 AEs, with no grade 5 AEs reported; no significant difference was observed between the two groups (23.9% vs. 33.9%, P = 0.220) (**Table 4**).

**Table 4:** Treatment-emergent adverse events.

	Any grade (cases)		P	Grades 3–4 (cases)		P
	Triple therapy (n = 71)	Quadruple therapy (n = 53)		Triple therapy (n = 71)	Quadruple therapy (n = 53)	
Total, n (%)	60 (84.50)	48 (90.56)	0.319	17 (23.94)	18 (33.96)	0.220
Abnormal liver function, n (%)	36 (50.70)	27 (50.94)	0.979	10 (14.08)	8 (15.09)	0.875
Fever, n (%)	33 (46.48)	28 (52.83)	0.484	0 (0.00)	0 (0.00)	–
Abdominal pain, n (%)	30 (42.25)	24 (45.28)	0.736	4 (5.63)	2 (3.77)	–
Anorexia, n (%)	23 (32.39)	16 (30.19)	0.794	0 (0.00)	0 (0.00)	–
Proteinuria, n (%)	22 (30.99)	17 (32.08)	0.897	7 (9.85)	5 (9.43)	0.937
Fatigue, n (%)	21 (29.58)	15 (28.30)	0.877	0 (0.00)	0 (0.00)	–
Thrombocytopenia, n (%)	20 (28.17)	13 (24.53)	0.650	8 (11.27)	6 (11.32)	0.993
Leukopenia, n (%)	19 (26.76)	13 (24.53)	0.779	0 (0.00)	0 (0.00)	–
Rash, n (%)	18 (25.35)	15 (28.30)	0.713	0 (0.00)	2 (3.77)	0.181
Hand-foot skin reaction, n (%)	16 (22.53)	14 (26.42)	0.618	0 (0.00)	0 (0.00)	–
Hypothyroidism, n (%)	15 (21.13)	13 (24.53)	0.654	0 (0.00)	0 (0.00)	–
Diarrhea, n (%)	15 (21.13)	12 (22.64)	0.840	0 (0.00)	0 (0.00)	–
Hypertension, n (%)	13 (18.31)	11 (20.75)	0.733	0 (0.00)	0 (0.00)	–
Vomiting, n (%)	10 (14.08)	8 (15.09)	0.875	0 (0.00)	0 (0.00)	–
RCCEP, n (%)	5 (7.04)	9 (16.98)	0.084	0 (0.00)	0 (0.00)	–
Immune-related pneumonitis, n (%)	2 (2.82)	6 (11.32)	0.072	0 (0.00)	2 (3.77)	0.181

*P* values were calculated using a two-sided χ^2^ test; when the sample size was small, and the expected frequencies were <5, Fisher’s exact test was used. RCCEP, Reactive cutaneous capillary endothelial proliferation.

## Discussion

HCC is among the most lethal malignancies worldwide, with particularly poor outcomes in advanced-stage disease. Although curative-intent treatments, including surgical resection, liver transplantation, and local ablation, can achieve favorable outcomes in early-stage HCC, most patients present with intermediate or advanced disease that precludes these options, highlighting the urgent need for more effective systemic and combination therapies (19). With the increasing adoption of multimodal treatment strategies, TACE-HAIC with systemic agents such as TKIs and PD-1 inhibitors represents a promising therapeutic approach for uHCC (20, 21).

In the present study, PSM was used to compare the clinical outcomes of TACE-HAIC+lenvatinib, without or with PD-1 inhibitors, in patients with uHCC. After balancing baseline characteristics, we comprehensively evaluated OS, PFS, and safety outcomes to determine whether the addition of immunotherapy provides incremental clinical benefit. After PSM, the quadruple-therapy group demonstrated a substantial survival advantage, with a median OS of 25.0 months compared with 11.0 months in the triple-therapy group. Median PFS was also prolonged (12.1 vs. 8.6 months). These findings are consistent with previous studies. Huang et al. reported a median OS of 21 versus for TACE-HAIC combined with TKI+PD-1 inhibitor (20).Similarly, Yuan et al. demonstrated a markedly improved median PFS of 14.8 months for TACE-HAIC based combination therapy in patients with HCC and portal vein tumor thrombosis (7).

In our study, the median OS of the quadruple therapy group reached 25.0 months, nearly 2.3-fold longer than the 11.0 months observed in the triple therapy group, whereas the median PFS only extended by approximately 3.5 months (12.1 vs 8.6 months). This striking mismatch between OS and PFS gain strongly reflects this typical immunotherapeutic curve tail effect. The markedly greater improvement in median OS relative to modest PFS prolongation is largely attributable to the unique “tail effect” of immune checkpoint inhibitors. Unlike traditional cytotoxic agents, PD-1 inhibitors can establish long-term anti-tumor immune memory and sustain durable tumor control even after radiographic disease progression. This sustained anti-tumor immunity continuously delays death events, thereby generating a long flat tail on the OS survival curve and driving a far more prominent OS benefit compared with PFS, which only captures the time to initial tumor progression (22, 23).

The observed survival benefits of the quadruple regimen may be explained by synergistic interactions among its individual components (24). TACE-HAIC delivers high local concentrations of chemotherapeutic agents and induces tumor ischemia through arterial embolization, resulting in direct cytotoxic effects. Lenvatinib, a multitarget TKI, inhibits tumor angiogenesis and proliferation (25). PD-1 inhibitors restore antitumor immunity by reversing T-cell exhaustion and reactivating immune surveillance (26). Locoregional therapy–induced tumor antigen release may enhance tumor immunogenicity and thereby create favorable conditions for immunotherapy. Concurrently, TKI-mediated normalization of the tumor microvasculature may facilitate immune cell infiltration, while PD-1 blockade further alleviates immunosuppression. Together, these effects may generate a systemic antitumor response (27–29), providing a mechanistic basis for the observed improvements in PFS and OS (29).

Several aspects of our findings warrant further consideration. First, our cohort included a substantial proportion of patients with Barcelona Clinic Liver Cancer Stage C disease and portal vein invasion, reflecting a higher tumor burden than that reported in most pivotal trials. Despite this, quadruple therapy demonstrated significant survival benefits. Second, although 43.6% of patients in the triple-therapy group subsequently received PD-1 inhibitors after disease progression, their OS remained inferior. This observation is consistent with findings by Xu et al., who reported superior OS with concurrent administration of lenvatinib and PD-1 inhibitors compared with sequential use (25). These data suggest that early integration of immunotherapy is critical for maximizing clinical benefit. Third, although the ORR based on mRECIST was comparable between groups (86.0% vs. 88.4%), the ORR based on RECIST v1.1 was numerically higher in the quadruple-therapy group (46.5% vs. 32.6%). This discrepancy likely reflects the greater sensitivity of mRECIST in detecting intertumoral necrosis induced by locoregional therapies.

Regarding safety, the overall incidence of TEAEs was comparable between the two groups (84.5% vs. 90.6%; P = 0.319), and no grade 5 AEs were observed, indicating an acceptable safety profile. The quadruple regimen did not significantly increase the incidence of grade 3–4 AEs compared with triple therapy (33.9% vs. 23.9%, P = 0.220), which is consistent with a previous report (21). However, a numerically higher incidence of immune-related pneumonitis was observed in patients receiving quadruple therapy (11.3% vs. 2.8%, P = 0.072). Although this difference did not reach statistical significance, the trend warrants clinical attention given the potentially serious nature of immune-related pneumonitis. Therefore, careful monitoring, early detection, and prompt management of immune-related adverse events are essential when administering quadruple therapy to ensure treatment safety.

Overall, these findings indicate that incorporating PD-1 inhibitors into TACE-HAIC+TKI regimens may provide additional survival benefits, particularly by enhancing durable disease control. The advantages of early immunotherapy initiation may be especially pronounced in patients with aggressive tumor biology, in whom sustained immune-mediated tumor suppression is critical (30, 31).

Nonetheless, our study has several limitations. First, this single-center retrospective analysis included a relatively small sample of 43 matched pairs, which may compromise the generalizability of our findings. Second, constrained by the retrospective design and limited sample size, we were unable to conduct formal sensitivity analyses (such as rank-preserving structural failure time models or inverse probability of censoring weighting) to adjust for biases induced by treatment crossover. As a result, the survival benefit conferred by upfront quadruple therapy may have been underestimated. Third, despite the application of PSM, residual confounding from unmeasured covariates could not be fully eliminated. Fourth, the use of multiple PD-1 inhibitors introduced treatment heterogeneity that could not be fully evaluated. Finally, although we performed exploratory subgroup analyses, no statistically significant survival differences between quadruple and triple therapy were observed across all strata. Large-scale prospective multicenter randomized controlled trials are warranted to validate our current findings, further explore heterogeneity in treatment efficacy across subgroups stratified by BCLC stage, PVTT status and other key clinical covariates, and identify patient populations most likely to derive survival benefits from quadruple therapy.

## Conclusion

The addition of PD-1 inhibitors to TACE-HAIC+lenvatinib was associated with significantly improved OS and PFS in patients with uHCC, with an acceptable safety profile. These findings suggest that the quadruple regimen may represent a promising first-line treatment strategy for uHCC. Further large-scale clinical trials are required to corroborate these preliminary findings.

## Data Availability

The raw data supporting the conclusions of this article will be made available by the authors, without undue reservation.

## Supplementary material

Supplementary Figure 1 Subgroup analyses for OS comparing quadruple therapy versus triple therapy after PSM. OS, Overall survival; PSM, Propensity score matching.
