# Supplementary material for: TACE-HAIC plus TKIs with/without PD-1 inhibitors for unresectable hepatocellular carcinoma: a propensity score matching study
Source: Front Oncol. 2026 Aug 14;16:1874177. doi: 10.3389/fonc.2026.1874177 (PMC13521848; doi:10.3389/fonc.2026.1874177)
Supplement: Supplementary file 1 [file Table1.docx]

Table S1. Multivariate analysis of factors related to RFS.

| PD-1 inhibitors | Quadruple therapy (n = 53) |
| --- | --- |
|  |  |
| Camrelizumab, n (%) | 29(54.7) |
| Tislelizumab, n (%) | 24(45.3) |
