# Supplementary material for: TACE-HAIC plus TKIs with/without PD-1 inhibitors for unresectable hepatocellular carcinoma: a propensity score matching study
Source: Front Oncol. 2026 Aug 14;16:1874177. doi: 10.3389/fonc.2026.1874177 (PMC13521848; doi:10.3389/fonc.2026.1874177)
Supplement: Supplementary file 2 [file Table2.docx]

Table S2. Subsequent treatment after disease progression.

| Subsequent treatment | Triple therapy  (n = 71) | Quadruple therapy  (n = 53) | P |
| --- | --- | --- | --- |
| Additional immunotherapy, n (%) |  |  | <0.001 |
| Yes | 31 (43.66) | 0 (0.00) |  |
| No | 40 (56.34) | 53 (100.00) |  |
| Immunotherapy switching, n (%) |  |  | 0.145 |
| Yes | 0 (0.00) | 7 (13.21) |  |
| No | 71 (100.00) | 46 (86.79) |  |
| Targeted therapy switching, n (%) |  |  | 0.733 |
| Yes | 13 (18.31) | 11 (20.75) |  |
| No | 58 (81.69) | 42 (79.25) |  |
| Additional local therapies, n (%) |  |  | 0.941 |
| Yes | 21 (29.58) | 16 (30.19) |  |
| No | 50 (70.42) | 37 (69.81) |  |

*P* values were calculated using a two-sided χ2 test; when the sample size was small, and the expected frequencies were <5, Fisher’s exact test was used.
